# Degradation of Carbon Nanotube Array Thermal Interface Materials through Thermal Aging: Effects of Bonding, Array Height, and Catalyst Oxidation

**DOI:** 10.1021/acsami.1c05685

**Published:** 2021-06-23

**Authors:** Andreas Nylander, Josef Hansson, Torbjörn Nilsson, Lilei Ye, Yifeng Fu, Johan Liu

**Affiliations:** † Electronics Materials and Systems Laboratory, Department of Microtechnology and Nanoscience (MC2), Chalmers University of Technology, SE-412 58 Göteborg, Sweden; ‡ SHT Smart High-Tech AB, Kemivägen 6, 412 58 Göteborg, Sweden

**Keywords:** carbon nanotubes, thermal interface materials, reliability, thermal cycling, XPS

## Abstract

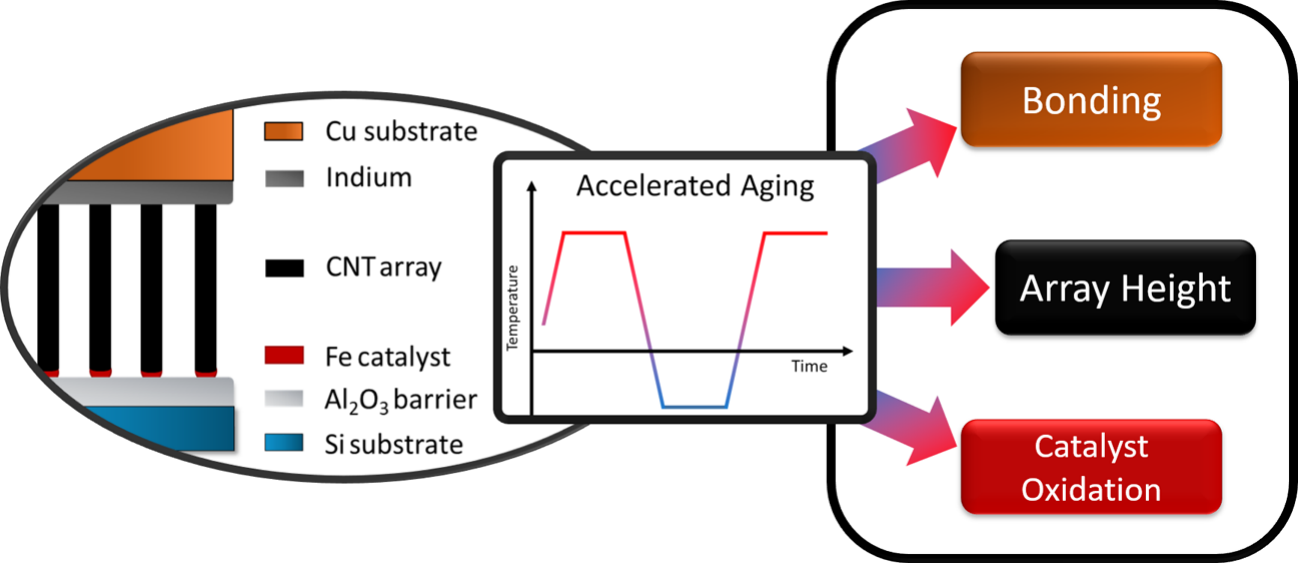

Carbon nanotube (CNT) array thermal interface materials (TIMs) are promising candidates for high-performance applications in terms of thermal performance. However, in order to be useful in commercial applications, the reliability of the interfaces is an equally important parameter, which so far has not been thoroughly investigated. In this study, the reliability of CNT array TIMs is investigated through accelerated aging. The roles of CNT array height and substrate configuration are studied for their relative impact on thermal resistance degradation. After aging, the CNT catalyst is analyzed using X-ray photoelectron spectroscopy to evaluate chemical changes. The CNT-catalyst bond appears to degrade during aging but not to the extent that the TIM performance is compromised. On the other hand, coefficient of thermal expansion mismatch between surfaces creates strain that needs to be absorbed, which requires CNT arrays with sufficient height. Transfer and bonding of both CNT roots and tips also create more reliable interfaces. Crucially, we find that the CNT array height of most previously reported CNT array TIMs is not enough to prevent significant reliability problems.

## 1 Introduction

Thermal interface materials (TIMs) are used to enhance heat transfer over an interface between two surfaces. The TIM conforms to the microscopic surface roughness of the mating surfaces and fills out voids that would otherwise be formed,^1^ thereby increasing the effective heat transfer. Today, TIMs are predominantly based on particle laden polymers (PLPs) which consist of thermally conductive filler particles suspended in a polymer matrix. However, it is difficult to achieve high thermal conductivity at low filler fractions, and increased filler fraction decreases the conformability of the TIM, limiting the total effectiveness of PLP TIMs. It is possible to use solder-based TIMs when the thermal performance is critical, but this might pose other challenges in terms of thermomechanical reliability.^2^

Carbon nanotubes (CNTs) have attracted attention for thermal applications due to their high thermal conductivity of up to 3000 W/mK.^3−5^ However, while there has been a lot of research on CNTs as filler in PLPs, the thermal conductivity of such composites has remained well below what would be expected from a rule of mixtures.^6,7^ This is mainly due to CNT matrix thermal boundary resistances and phonon dampening in the CNTs by the surrounding matrix.^8,9^

This problem can be avoided by using arrays of vertically aligned CNTs as TIMs. CNT array TIMs allow each CNT to span from one surface to the other, eliminating internal boundary resistances within the TIM. Grown CNT arrays are generally relatively sparse, with a filling factor less than 10%^10^ but still have an effective thermal conductivity on the order of 10–100 W/mK,^11^ significantly higher than commercial PLP TIMs. Since CNTs have high flexibility,^12^ CNT arrays can conform to the mating surfaces and achieve low thermal interface resistances.^13,14^

The most simple CNT array TIM consists of a CNT array grown on a Si substrate, pressed against an opposing substrate.^13^ However, uneven CNT length causes only the longest CNT to contact, with a large fraction of the CNTs not reaching the opposing surface.^15^ To solve this problem, a bonding agent that partially penetrates the CNT array can be used, which can be either polymer-based^16−18^ or metal-based.^15,19,20^ It is also often unfeasible to synthesize CNT arrays directly on one of mating surfaces. For instance, CNT array synthesis requires temperatures that exceed the maximum allowed temperature for complementary metal oxide semiconductor compatibility^14,21^ and thus cannot be done directly on an active chip. In this case, the transfer of the CNT array is required, by bonding the CNT array top side, removing the growth substrate, and subsequently bonding the bottom side of the array to another substrate.^22^

Since CNT arrays are relatively sparse, each CNT can flex independently in the *x*–*y* plane, potentially allowing for a good mechanical decoupling between the mating surfaces, reducing thermal stresses due to mismatch in the coefficient of thermal expansion (CTE) of the two surfaces. It is expected that CNT array TIMs should be able to combine excellent thermal performance with mechanical decoupling and thus good reliability. However, while there are plenty of studies on the thermal performance, the reliability of CNT array TIMs has so far been neglected.^1^

In our previous study, the effect of thermal cycling on polymer-bonded CNT array interfaces was investigated, and the results showed that the thermal interface resistance quickly increased by an order of magnitude.^23^ The increase, as well as the fact that several samples in the study completely delaminated, shows that the reliability of CNT array TIMs cannot be taken for granted. The CNT arrays were delaminated at the CNT roots, and further analysis indicated that oxidation of the iron catalyst at the interface between CNTs and the substrate may play a role in the degradation. In addition, the length of the CNTs was around 15 μm, which is in line with most previous studies, but which might not be long enough to absorb the CTE mismatch strain without enough tension to uproot the CNTs at the edges. However, without further investigation, it is difficult to ascertain to what extent both of these components matter for the overall reliability and what constraints there are on the design of reliable CNT array TIMs.

In this work, metal-bonded CNT array TIMs were subjected to accelerated aging by thermal cycling, and the effects of both the catalyst oxidation and the CNT array height were investigated in order to determine their relative impact on reliability. We find an exponential relationship between the CNT array height and thermal interface resistance degradation during temperature cycling. Chemical analysis found that oxidation of the catalyst is present but does not appear to dramatically influence the thermal interface resistance in the absence of CTE mismatch. Nonetheless, the CNT/catalyst interface is found to be the weakest link, and transfer and double bonding of CNT arrays allow for reliable TIMs using shorter CNTs.

## 2 Materials and Methods

The CNT arrays were fabricated by chemical vapor deposition (CVD) in a base-growth configuration on silicon substrates.^24^ The 8 × 8 mm Si substrates were prepared with a catalyst by deposition of 10/1 nm Al_2_O_3_/Fe using e-beam evaporation (AVAC HVC 600). The CVD system used was a commercial cold wall CVD system (Aixtron Black Magic). The reaction took place at low pressure in a vacuum chamber on a graphite heater.

First, the samples were annealed at 500 °C under a flow of 837 sccm H_2_ for 3 min. This was followed by a growth step at 700 °C and 200 sccm of C_2_H_2_. The desired CNT array height was obtained by varying the growth step length accordingly.^25^

**Figure 1:**
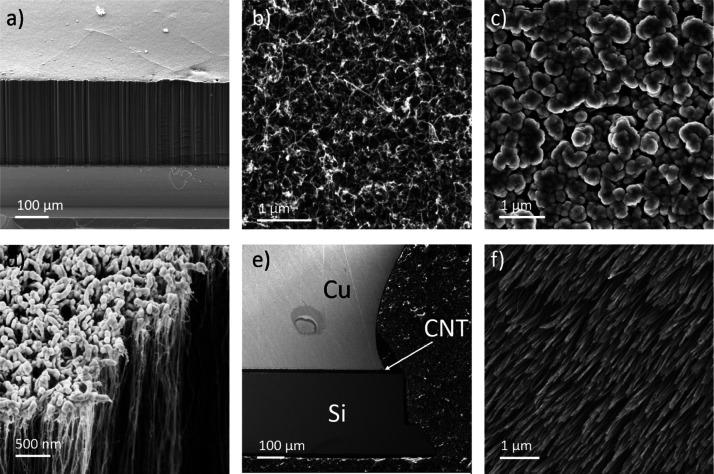
SEM image of the CNT array TIM components. (a) As-grown CNT array. (b) Top of the CNT array. (c) Top of the CNT array after metallization. (d) Side view of the CNT array after metallization. (e) Cross section of the assembled Si/Cu interface with the CNT array bonded in between. (f) Root side of the CNT array after delamination from the silicon growth substrate.

The CVD process produces uniform CNT arrays, such as the one seen in Figure 1a, which is an example of a 200 μm as-grown CNT array. Further details regarding the quality of the as-grown CNTs with a transmission electron microscopy (TEM) image (Figure S1), X-ray photoelectron spectroscopy (XPS) spectrum (Figure S2), as well as a Raman spectrum (Figure S3) are presented in the Supporting Information. After CVD synthesis, the CNT arrays were bonded to opposing surfaces using an indium-based transfer method.^22^ First, the exposed CNT tips were covered in a 10/20 nm thick layer of Ti/Au by sputtering. Figure 1b,c shows the top of the CNT array before and after sputtering, and Figure 1d shows a side view of a cross section of the metallized CNT array. As can be seen, the sputtering creates a coating which adheres to the top of the array but only penetrates in the order of 100 nm into the array. The mating surface was covered with a 10/20/1000 nm layer of Ti/Au/In.

After sputtering, the array was bonded to an opposing surface at a temperature of 200 °C and a pressure of 100 kPa. The In layer melted and soldered the two surfaces together. This bonding method results in interfaces with higher thermal resistance than some other state-of-the-art methods but is fast and highly scalable. As long as the degradation of the interface during thermal cycling occurs primarily on the CNT root side, the reliability behavior of the CNT array TIMs should be independent of the bonding method. During the bonding procedure, the interface compressed, the extent of which can be seen in Figure 1e. This shows a cross section of a CNT array bonded to Cu, obtained by epoxy encapsulation, cutting, and polishing using a standard procedure for electronics cross sectioning. The resulting bond line thickness of the interface was in the end only around 30% of the initial CNT array height. Nevertheless, during the study, the length of the CNTs is more important than the bond line thickness of the interface since the thermal transport will occur predominantly along the entire length of the CNT. For this reason, the CNT array height was used for comparison within the study rather than the bond line thickness.

**Figure 2:**
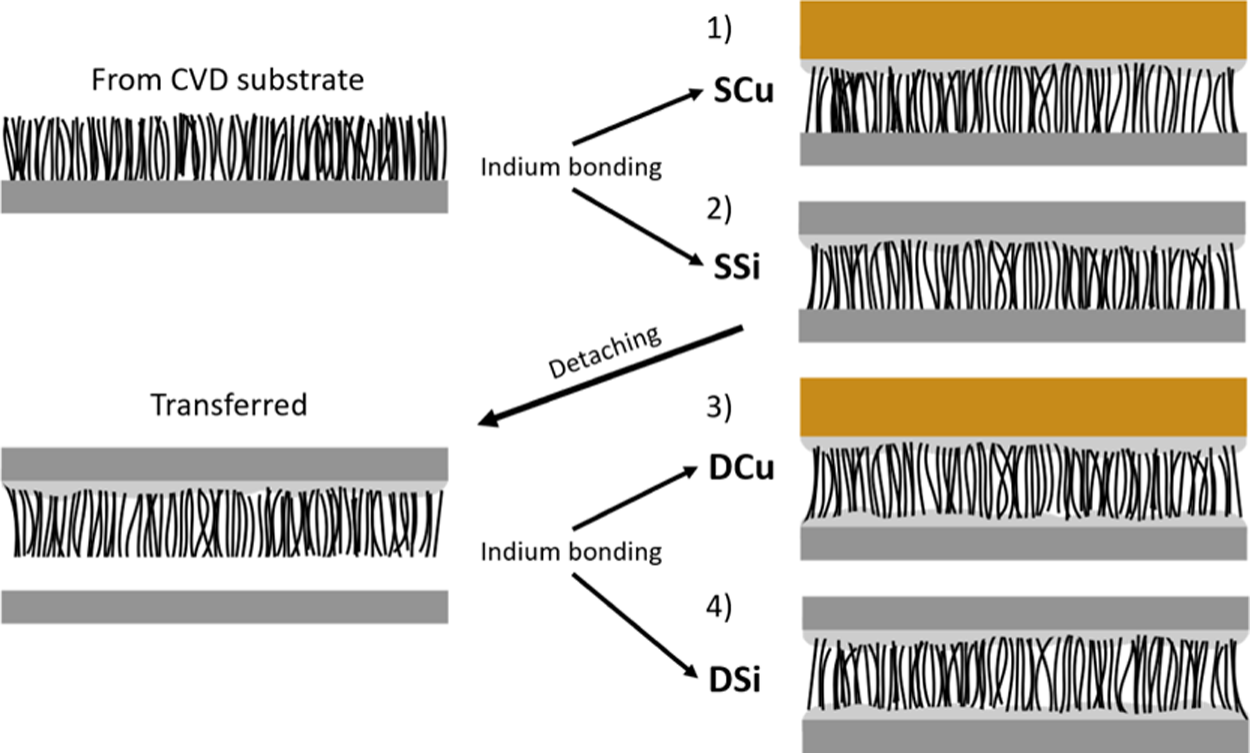
Assembly of different TIM structures. The CVD growth substrates were bonded to Cu (1) or Si (2) pieces, and some samples were further detached from the growth substrate and bonded to Cu (3) or Si (4) on the CNT root side.

In order to investigate the influence of CNT array height on the reliability, samples with four different array heights were investigated. Heights of 10, 50, 80, and 200 μm were prepared, corresponding to a CVD growth time of 5 s, 1 min, 2 min, and 5 min, respectively. All of these samples were bonded to a copper piece, 8 × 8 × 1 mm, and coated with standard electroless nickel immersion gold plating, corresponding to configuration SCu (single-bonded Si-CNT-Cu) in Figure 2.

Arrays of 80 μm long CNTs were further assembled into all four configurations seen in Figure 2. In configurations SCu and SSi (single-bonded Si-CNT-Si), the CNT growth substrate was directly bonded to a Si or Cu piece. Since the SSi interface is free from CTE mismatch, the effect of CTE mismatch on the interface degradation should correspond to the difference between SCu and SSi.

In the DCu and DSi configurations (double-bonded Si-CNT-Cu and Si-CNT-Si respectively), the CNT array was bonded to a Si surface, the growth substrate was removed, and a Cu or Si piece was bonded to the CNT roots. Figure 1f shows an image of the exposed CNT roots after delamination. The delamination was done by shearing the interface, which caused the bottom of the CNT roots to be dragged in one direction and aligned in a furlike pattern. By comparing the single- and double-bonded configurations, the difference between single-bonded and transferred CNT arrays was found. Among the samples in the SCu configuration, half the samples were bonded with the tips to the Cu side and half with the roots. In total, at least 10 samples of each configuration were fabricated and tested.

The thermal interface resistance of the samples was measured using the laser flash method (Netzsch LFA 447) employing the double layer plus contact resistance model.^26,27^ This technique approximates the CNT array in the interface as a contact resistance contribution, and by measuring the Si and Cu substrates separately and including their data in the model, the CNT array thermal interface resistance *R*_th_ can be extracted and isolated as equivalent to
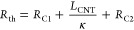
1where *R*_C1_ and *R*_C2_ are the CNT contact points of either side of the interface, *L*_CNT_ is the original height of the CNT array before compression during bonding, and κ is the effective thermal conductivity of the CNT array. As the thermal transport takes place along the entire length of the CNT, this equation uses the original CNT length before bonding instead of the bond line thickness of the interface, which otherwise is the case for conventional TIM types.

The samples were aged by thermal cycling using a LN2-connected thermal cycling oven (Despatch 400). The temperature profile was chosen according to JEDEC standard test condition “B”^23,28^ corresponding to a cycle between −55 and 125 °C, 10 min ramp rate, and 20 min holding time at each temperature extreme. This temperature range is wider than necessary for most real world applications but is a commonly used standard temperature cycling profile to ensure good reliability. The thermal interface resistance was measured before cycling as well as periodically during thermal cycling at approximately logarithmically increasing intervals.

In order to investigate the catalyst before and after aging, we used XPS (PHI 5000C) on the growth substrates after uprooting the CNT arrays for a full exposure of the catalyst structure. The XPS measurements were conducted with an Al filament, and the acquired spectra were calibrated by shifting the C 1s peak to coincide with the binding energy of adventius carbon at 284.8 eV. A Shirley-type background was accounted for during quantification of the spectrum data. Multiplex scans of the C 1s, Fe 2p, Al 2p, and O 1s were conducted and compared for their relative contributions. Furthermore, extensive peak fitting was done to reveal the individual contributions in the C 1s and Fe 2p peaks.

## 3 Results

### 3.1 CNT Array Height Influence

**Figure 3:**
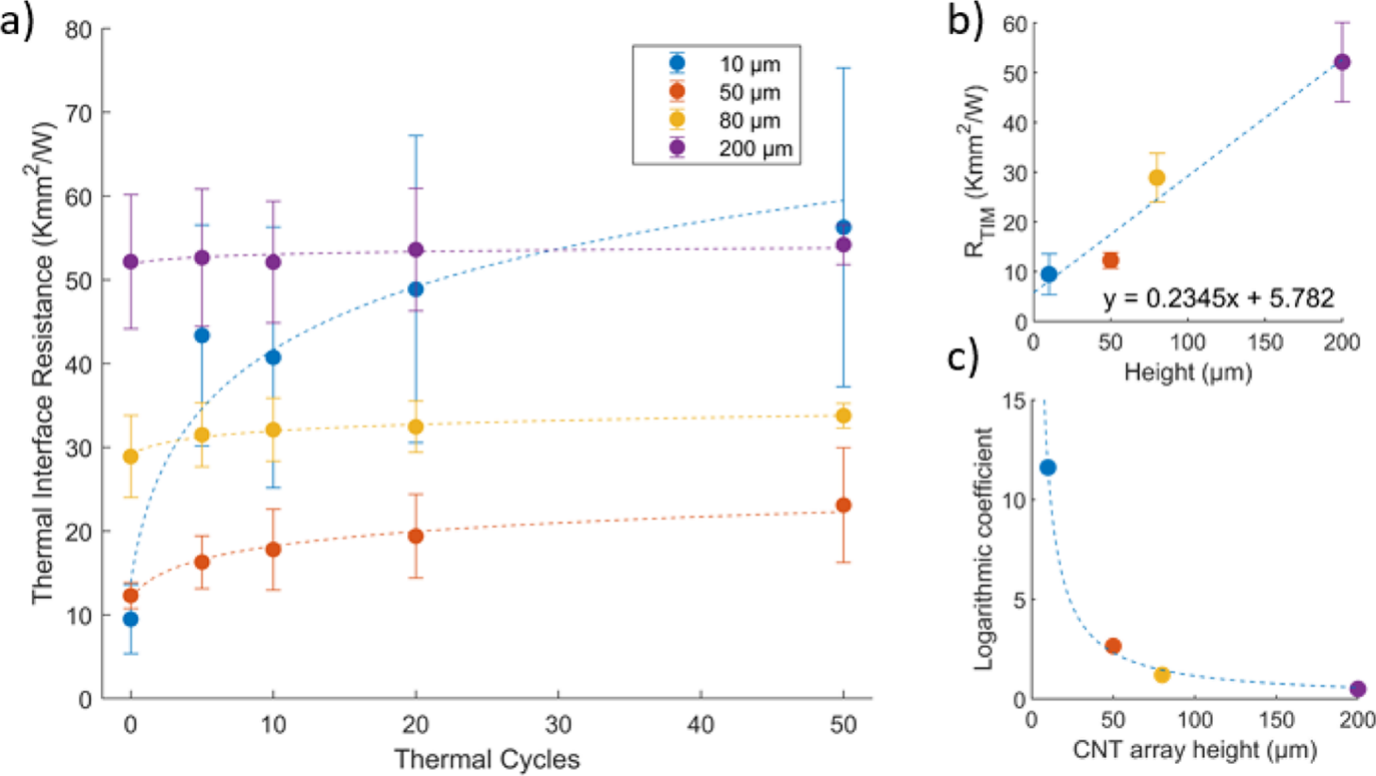
(a) Thermal interface resistance as a function of thermal cycles for different CNT array heights in SCu configuration. (b) Thermal interface resistance as a function of the CNT array height before thermal cycling. (c) Logarithmic coefficients of the fitted curves in (a) as a function of the CNT array height, with an exponential fitting.

Figure 3a shows the thermal interface resistance evolution during thermal cycling for 50 cycles, while Figure 3b shows the initial thermal interface resistance versus CNT array height. As it can be expected, the thermal interface resistance increases linearly with increasing height, although a slope coefficient implies an effective array thermal conductivity of only 4.3 W/mK, while previous measurements of CNT arrays produced by the same method have found an effective free standing thermal conductivity of around 70 W/mK.^23^ This implies that our bonding method does not manage to engage all CNTs in the array for effective heat transfer since the intertube heat transfer within the CNT array is negligible.^15,29^ Nonetheless, as seen in Figure 3a, there is a clear relationship between the thermal interface resistance degradation and the CNT array height during thermal cycling, with an immediate dramatic increase for the 10 μm samples and smaller increases with longer CNT arrays. Indeed, some 10 μm samples delaminated completely and had to be discarded.

Simple calculations can highlight why the reliability is severely compromised at a CNT array height of 10 μm. The displacement difference Δ*x*^30^ between the Cu and Si surfaces for the outermost corner in one thermal cycle is equal to
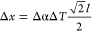
2where Δα is the CTE mismatch, Δ*T* is the temperature span, and  is the edge length of the interface (assuming square shaped interface). For an interface area of 8 × 8 mm with Si on the one side and Cu on the other, we get Δ*x* ≈ 14 μm (Δα = 14 ppm/K at 20 °C), which is already more than the entire CNT length. The lateral displacement Δ*x* between the substrates will act on the CNT in the array with a stretching S according to
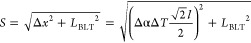
3where *L*_BLT_ is the bond line thickness of the interface. Assuming that *L*_BLT_ of the bonded interface is 30% of the original CNT array length, according to Figure 1e, the total strain for a 10 μm CNT array will be *S* = 14.3 μm under the described conditions. This would require the CNT lattice together with its contact points on either side of the interface to absorb a strain of 43%. As the CNT array is locked in an interface, the weakest point will determine the mechanical reliability, and experiments have demonstrated that our one-sided interfaces always delaminate on the catalyst side. However, even by removing the growth substrate, the CNT lattice will break at far lower strains, and experiments have demonstrated CNTs to exhibit as low as 3–5% elongation at break.^12^

While this poses an obvious problem, the majority of CNT array TIM research to date has concerned CNT arrays with heights in this order of magnitude.^1^ Thus, future research on CNT array TIM aimed applications with CTE mismatch, such as most TIM1 applications, should focus on CNT arrays with sufficient height to ensure reliability.

The thermal resistance during thermal cycling appears to roughly follow a logarithmic increase. The dashed lines in Figure 3a show fitted logarithmic curves, and Figure 3c shows the logarithmic coefficient of the curves as a function of the CNT array height. These coefficients in turn show a reciprocal relationship with the CNT array height. In practice, this means that the thermal interface resistance degradation quickly increases as the CNT array height decreases, suggesting a trade-off between thermal performance and reliability.

By combining the relationship between the CNT array height and thermal resistance degradation in Figure 3a with the logarithmic fitting in Figure 3c, we can derive an expression for the required CNT length *L*_CNT_, given an absolute performance degradation Δ*R*_th_ and a certain thermal cycle count *n* according to
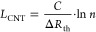
4

The constant *C* in the equation is extracted from the curve fitted data in Figure 3c and depends on the strain in the interface, how well the CNTs are bonded in the interface, and ultimately the crystallinity of the CNT lattice. By comparing reliability demands to thermal paste that is expected to double in regards to the thermal interface resistance after 1000 cycles,^31^ some conclusions can be made about the studied system. A state of the art CNT array TIM is, according to literature, expected to possess a resistance performance of at least 2 mm^2^K/W.^1^ Using these numbers in the case of the studied interface from Figure 3, we get *C* = 116.4 which in turn gives a required CNT length of 402 μm assuming Δ*R*_th_ = 2 mm^2^K/W and *n* = 1000.

### 3.2 Interface Configuration and CTE Influence

**Figure 4:**
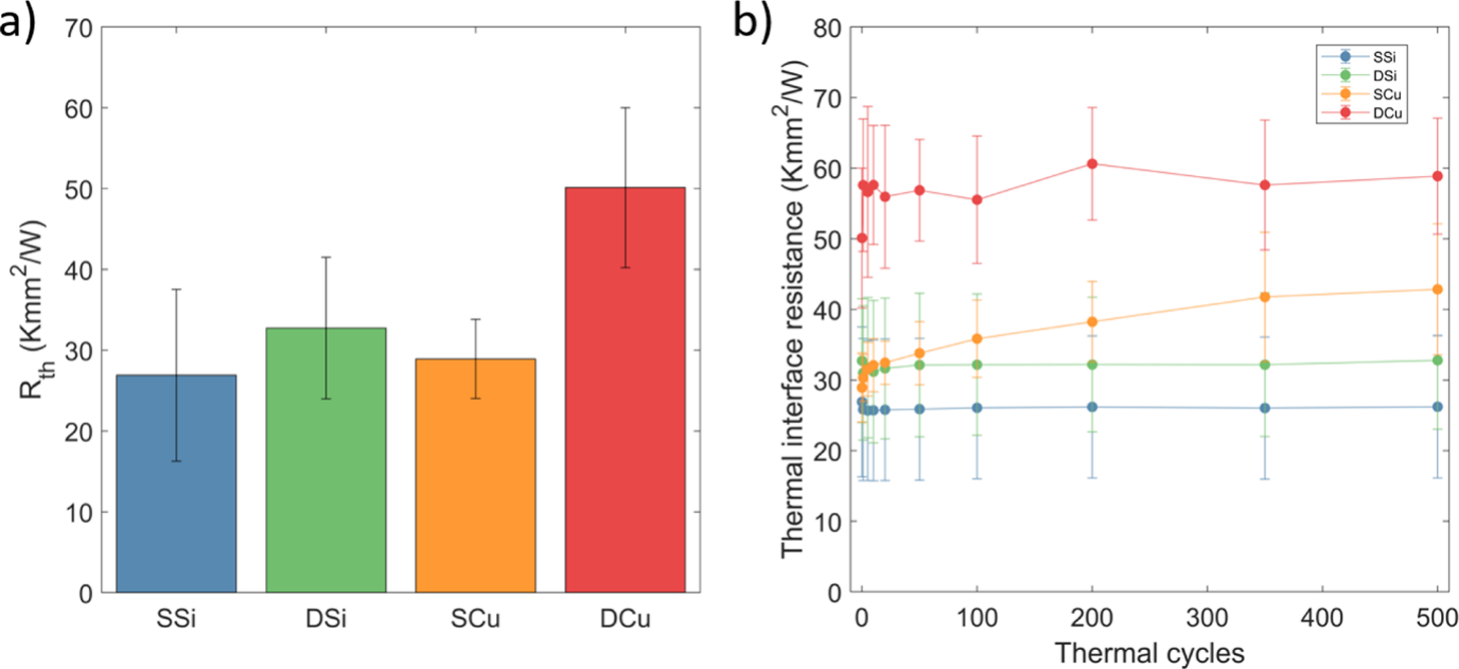
(a) Thermal interface resistance of all sample configurations before thermal cycling using 80 μm tall CNT arrays. (b) Thermal interface resistance evolution during thermal cycling. The error bar denotes the standard deviation between samples.

A CNT height of 80 μm was chosen for the investigation of the long-term cycling of the different sample configurations as it had a noticeable but not too severe degradation during the first 50 cycles for the SCu configuration. Figure 4a shows the initial thermal interface resistance for the different configurations and (b) the thermal cycling evolution over 500 cycles. The double-bonded configurations both exhibit a higher total thermal interface resistance, again consistent with only partial bonding, although the increase from SSi to DSi is much smaller than SSi to SCu, possibly due to differing surface roughness.

During thermal cycling, the thermal interface resistance is stable for both SSi and DSi configurations. The SCu configuration continues to increase continuously during the entire cycling, up to an overall increase of 50%, from 29.8 to 42.8 Kmm^2^/W after 500 cycles. The DCu configuration experienced an abrupt increase from 50.1 to 57.6 Kmm^2^/W after the first cycle but has no consistent increase during subsequent cycling.

The fact that both Si–Si configurations appear completely stable suggests that the thermal resistance increase seen in the SCu samples are mainly due to the CTE mismatch stress rather than catalyst degradation, which should otherwise appear in the SSi configuration. Additionally, comparing DCu with SCu shows that, at the same CNT height, transfer of the CNT array away from the growth substrate and bonding the roots can create a more reliable interface than the one utilizing the growth substrate. While the thermal interface resistance of DCu is still significantly higher than that of the aged SCu, with an optimized bonding method, it should be possible to create a high-performing and reliable interface.

### 3.3 CNT Catalyst Degradation

Previous studies concluded that the degradation of CNT array TIM interfaces either originated from the CTE mismatch between the mating substrates or a chemical change in the catalyst structure.^23^ The CNTs are attached to the growth substrate with the catalyst particles as anchor points through an intermediate iron carbide layer.^32^ Therefore, a chemical change could potentially release the CNTs from the growth substrate which would result in a TIM performance degradation and spontaneous interface delamination in the most severe cases.

**Figure 5:**
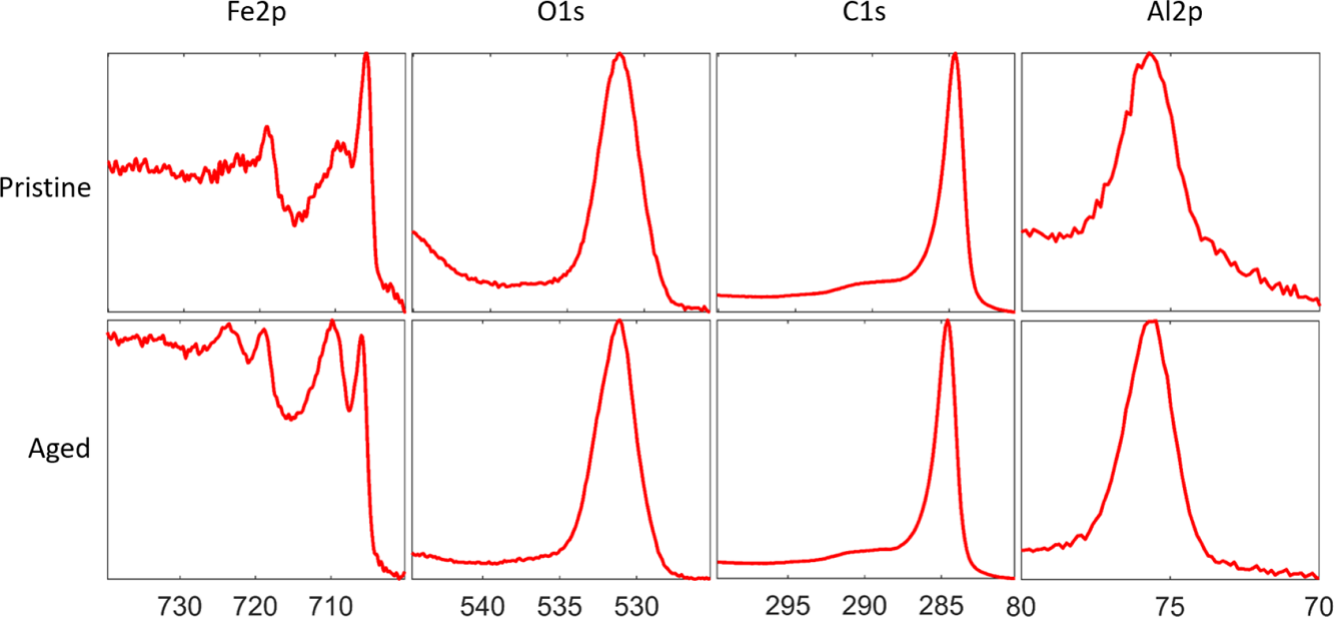
XPS spectrum peaks of Fe, O, C, and Al residues on the growth Si substrate after delamination of the CNT arrays. Top row depicts data from the pristine as-grown CNT arrays, while the bottom row depicts data from samples aged by thermal cycling for 500 cycles.

The elemental composition of the catalyst structure was analyzed using XPS before and after thermal cycling. This was done by forced delamination of pristine and aged CNT arrays and analysis of the remains left on the silicon growth substrate. Figure 5 shows the Fe 2p, O 1s, C 1s, and Al 2p peaks before and after aging.

**Figure 6:**
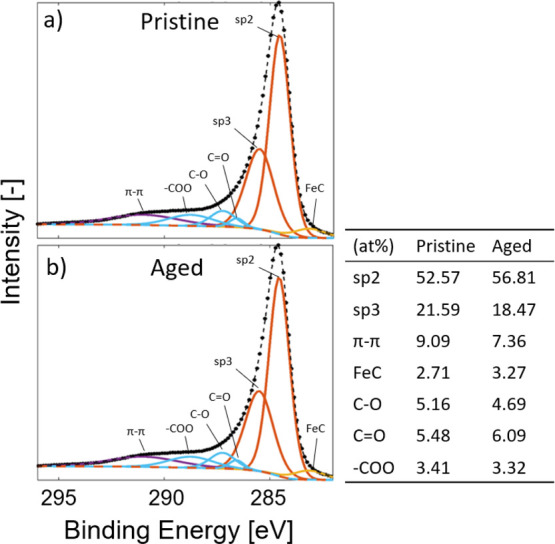
Detailed analysis and deconvolution of the C 1s XPS peak for (a) pristine and (b) aged samples together with the bond type composition.

**Figure 7:**
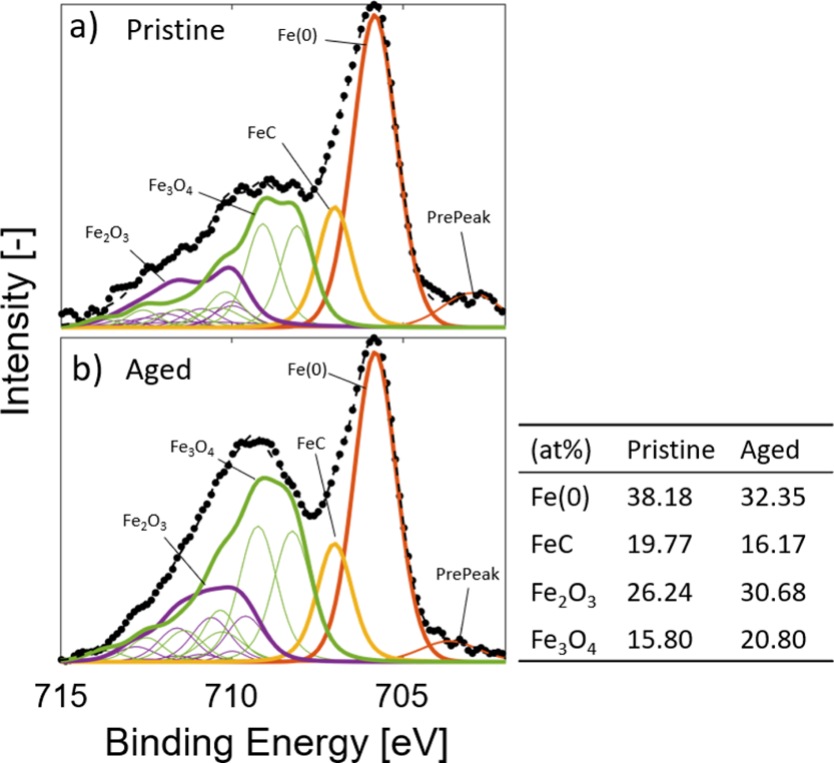
Detailed analysis and deconvolution of the Fe 2p XPS peak for (a) pristine and (b) aged samples together with the bond type composition. The background has been subtracted in order to facilitate comparisons.

The composition of elemental species on the sample surface was determined by comparing and weighing the relative area of each of the peaks accordingly.^33^Table 1 shows the determined composition ratio. The main change is a relative decrease in carbon and the corresponding increase for the other species present, with the iron contribution in particular showing a larger increase than what can be explained by a simple reduction in carbon. The appearance of the Fe 2p peak changed as expected, similar to a previous study;^23^ otherwise, there were no obvious changes in the peak shape. The elemental composition is consistent with a weakening between the iron catalyst nanoparticles and the CNTs. After aging, more iron and less carbon were left behind on the substrate after uprooting, explaining both the decrease in carbon, the relative increase of aluminum and oxygen, as well as the larger increase in iron. Further analysis was conducted by fitting the Fe 2p and C 1s peaks to their individual components. These results can be found in Figures 6 and 7.

**Table 1:** Elemental Composition of CNT Catalyst Residues as Determined from XPS Data in Figure 5

(wt %)	Fe 2p	O 1s	C 1s	Al 2p
pristine	0.12	4.68	93.25	1.95
aged	0.20	5.32	91.28	3.20

The C 1s peak seen in Figure 6 from before and after thermal aging of the CNT TIM samples was fitted and analyzed. Using reference studies on oxidized CNTs, it was concluded that the components of interest were primarily sp^2^ and sp^3^ bonded carbon,^33^ FeC,^34^ and a number of oxygen functional groups (C–O, C=O, −COO).^35,36^ According to the results in Figure 6, the chemical composition of the C 1s peak is more or less unaltered with some insignificant differences. The most significant change is the FeC component, which increased by 20% after aging. However, considering that the total iron content from Table 1 has increased by 67% while the carbon content has decreased, a relative increase in the FeC component of the C 1s peak is expected.

For the Fe 2p peak, a similar analysis is shown in Figure 7. The included components were elemental iron (Fe(0)), iron carbide (FeC), a “pre-peak” associated with defects in the iron phase,^37^ as well as the oxides Fe_2_O_3_ (alpha and gamma phase—Fe^3+^) and Fe_3_O_4_ (Fe^2+^, Fe^3+^).^38^ The peak fit disregarded FeO (Fe^2+^) due to its instability at room temperature that causes it to combine into Fe_3_O_4_.^39^ In order to facilitate comparison, the background was subtracted and the fitting was limited to the Fe 2p_1_2 part of the total peak. The subsequent Fe 2p_3_2 part, which can be seen as the second set of dual peaks in the Fe 2p part of Figure 5, contains similar information.

The differences between the pristine and aged samples are a decrease in metallic iron and iron carbide and an increase in iron oxides. This shows an obvious oxidation of the iron component, which is a likely contributor to the weakening of the bond between the iron catalyst and the CNTs. The decrease in carbide is also indicative of degraded bonding^40^ and is also consistent with the lower degree of carbon compared to the other elements, as seen in Table 1. Overall, the XPS analysis shows that aging causes oxidation of the iron catalyst, which in turn weakens the bond between the catalyst and the CNTs. However, the magnitude of this degradation does not in itself appear to cause significant reliability issues. If it had, the SSi sample configuration should exhibit some sort of degradation in comparison with the DSi configuration, which does not appear to be the case. A previous study^23^ found a small thermal boundary resistance increase of about 1 Kmm^2^/W at the CNT roots after 30 thermal cycles. If this had been a linear trend, the thermal resistance increase over 500 cycles should be on the order of 10 Kmm^2^/W, which should be clearly visible in the collected data. Thus, the previously found effect might not be linear with respect to thermal cycling, and the total magnitude might be lower than the accuracy within our measurements. However, regardless of the reliability concerns, this phenomenon should be further studied as it might have implications for transfer processes of CNT arrays. If sufficient weakening of the CNT catalyst bond can be achieved, delamination from the growth substrate could be achieved without the need of shearing, which misaligns the array in the process.

## 4 Discussion

With improved CNT array quality and bonding methods, CNT array TIMs with much lower total thermal interface resistance can be expected. In this case, the catalyst degradation effect could become a limiting factor. However, the most important factor for the reliability of CNT array TIMs appears to be to match the CNT array height with the application CTE mismatch. This is something that is currently rarely taken into account within the literature. Table 2 shows previously reported single-bonded or dry contact CNT array TIMs together with the respective opposing substrate, CTE mismatch, CNT array height, and thermal interface resistance *R*_th_, with the CNT array TIMs used in the height study as a comparison. As can be seen, the vast majority of previously published CNT array TIMs have CNT array heights in the range of 10–50 μm.

**Table 2:** Previous CNT Array TIM Reports with Si-Grown CNT Arrays toward an Opposing Substrate

opposing substrate	CTE mismatch (ppm/K)	CNT array height (μm)	*R*_th_ (Kmm2/W)	references
Ag	15	25	7	Cola et al.^14^
Ag	15	15	15.8	Cola et al.^13^
Ag	15	40	8	Amama et al.^41^
Ag	15	30	1.7	Cross et al.^19^
Ag	15	20	14	Hodson et al.^42^
Ag	15	15	4.6	Taphouse et al.^43^
Ag	15	10	4.9	Taphouse et al.^17^
Al	20	10	7	Zhang et al.^44^
Al	20	10	14.6	Gao et al.^45^
Al	20	28	12	Panzer et al.^15^
Cu	14	13	19	Xu and Fisher^46^
Cu	14	100	10	Lin et al.^47^
Cu	14	10	1.4	Ni et al.^16^
glass	8.5	7	11	Tong et al.^20^
glass	8.5	10	1	Tong et al.^20^
Ni	10	50	8	Liu et al.^48^
Cu	14	10	9.5	this work
Cu	14	50	12.3	this work
Cu	14	80	29	this work
Cu	14	200	52	this work

As previously mentioned, in order to prevent degradation of more than 2 Kmm2/W, a CNT height of 402 μm is required, higher than any published CNT array TIM to date. There is little relation between the thermal interface resistance and the CNT height when comparing between reports, but our own measurements show a clear increase in resistance with longer CNTs, highlighting the challenge of combining reliability and performance in CNT array TIMs. Indeed, based on reported values, every single report mentioned in Table 2 would have unacceptably high degradation during thermal cycling according to our model.

The thermal cycling profile used here represents significantly harsher temperature extremes than normal applications, and the areal size of the interface will influence the reliability behavior of the interface. Thus, it is possible that shorter CNT array TIM could create adequate interfaces for many applications. At the same time, real application of CNT array TIMs in microelectronic devices would experience uneven temperature gradients coming from the many hot spots present, which accounts for nonideal aging. Nevertheless, it should be noted that the thermal interface resistance increases found here are noticeable already after 50 cycles and that even relatively long 80 μm CNT arrays exhibited a significant resistance increase over the full 500 cycles.

Our results indicate that in order to create reliable CNT array TIMs, the CNT array height needs to be taken into consideration and that the heights common in the literature may be insufficient. Commercially viable solutions will require longer CNTs, transferred and double bonded arrays, or both.

## 5 Conclusions

We have investigated parameters influencing the reliability of CNT array TIMs. The reliability issues for CNT array TIMs appear to arise from insufficient absorption of CTE mismatch strain. While CNTs are flexible and can mechanically decouple the surfaces, CNTs of inadequate height will experience large tensile stresses and delaminate from the CNT root side. This is especially problematic since most CNT array TIMs utilize CNT array heights of 10–30 μm, which is far from guaranteed to result in reliable interfaces.

While signals of catalyst oxidation were found, no clear connection between the reliability and oxidation could be established. However, the catalyst-CNT connection is much weaker than bonded CNT tips, and transfer from the growth substrate and bonding of the CNT roots can improve the strength of the interface, allowing for reliability at lower CNT heights than otherwise possible.

The obtained data from this study were analyzed and used to derive a mathematical relation that can be used to calculate the required CNT array length for a given resistance degradation. This model is dependent on a constant that is directly related to the accumulated strain applied to the CNTs in the interface. By finding the constant associated with other interface configurations, this model can be adopted for other CNT array TIMs.

## Supporting Information Available

TEM imaging of the CNTs after growth showing the catalyst particles attached to the CNT roots; XPS analysis of the CNT array tips; and Raman analysis of the CNTs that highlights the quality of grown CNTs (PDF)
