# Supplementary material for: Degradation of Carbon Nanotube Array Thermal Interface Materials through Thermal Aging: Effects of Bonding, Array Height, and Catalyst Oxidation
Source: ACS Appl Mater Interfaces. 2021 Jun 23;13(26):30992–1000. doi: 10.1021/acsami.1c05685 (PMC8289226; doi:10.1021/acsami.1c05685)
Supplement: Supplementary file 1 — am1c05685_si_001.pdf [file am1c05685_si_001.pdf]

Supporting information
Degradation of Carbon Nanotube Array Thermal Interface Materials Through Thermal Aging: Effects of Bonding, Array Height and Catalyst Oxidation

Andreas Nylander,[†,¶] Josef Hansson,[†,¶] Torbjörn Nilsson,[†] Lilei Ye,[‡] Yifeng Fu,[†] and Johan Liu*,[†]

†*Electronics Materials and Systems Laboratory, Department of Microtechnology and Nanoscience (MC2), Chalmers University of Technology, SE-412 58 Göteborg, Sweden*

‡*SHT Smart High-Tech AB, Kemivägen 6, 412 58, Göteborg, Sweden. Present address: Chalmers Industriteknik, SE-412 58 Göteborg, Sweden*

¶*Equally contributing authors*

E-mail: johan.liu@chalmers.se

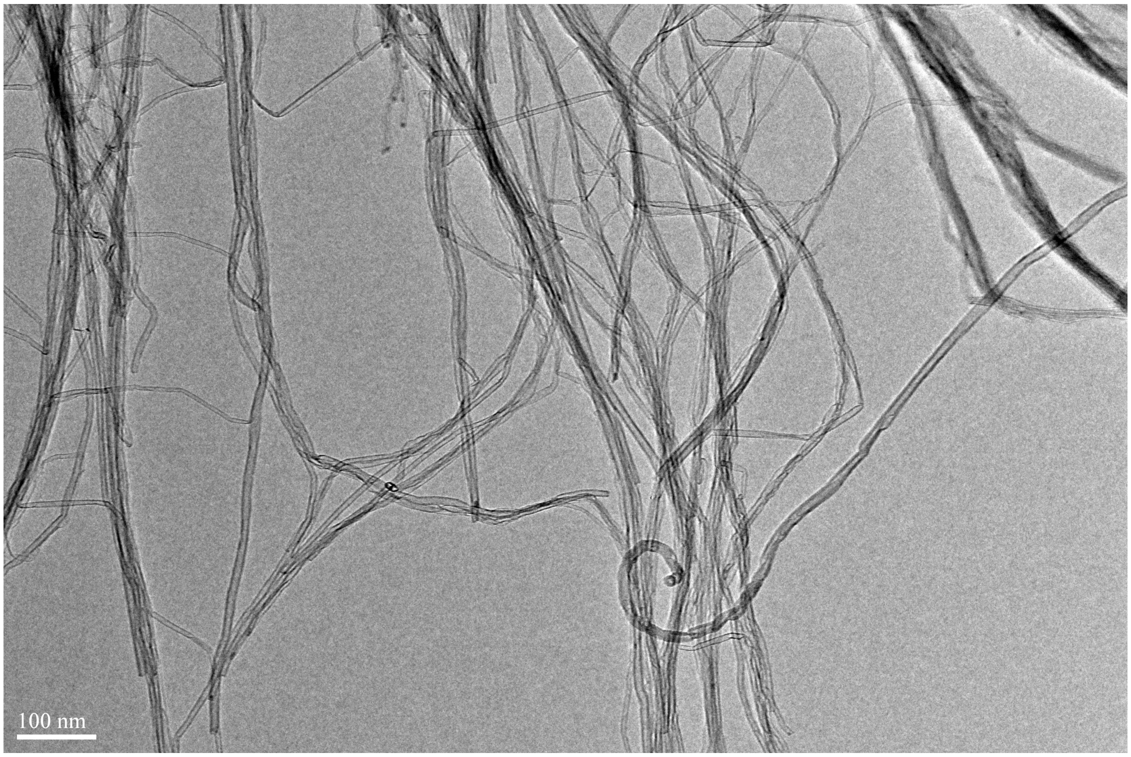

Figure S1: Transmission electron microscope image of the CNTs obtained from the CVD growth which exposes the catalyst at the CNT roots.

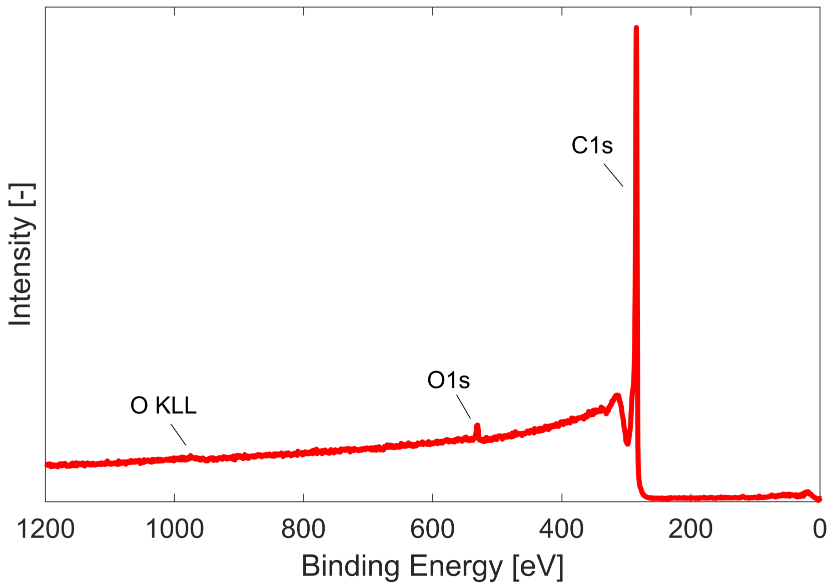

Figure S2: Chemical analysis of the top side of the CNT array after growth using XPS revealing the presence of carbon and oxygen on the CNT tips.

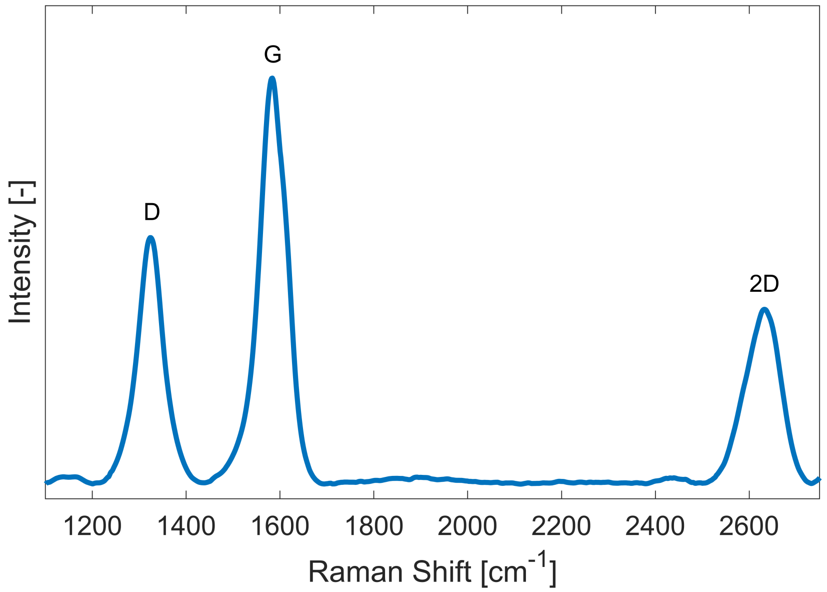

Figure S3: Chemical analysis of the grown CNTs using Raman spectroscopy highlighting the D, G and 2D peaks.
